# estMOI: estimating multiplicity of infection using parasite deep sequencing data

**DOI:** 10.1093/bioinformatics/btu005

**Published:** 2014-01-17

**Authors:** Samuel A. Assefa, Mark D. Preston, Susana Campino, Harold Ocholla, Colin J. Sutherland, Taane G. Clark

**Affiliations:** ^1^London School of Hygiene and Tropical Medicine, WC1E 7HT, London, UK, ^2^Wellcome Trust Sanger Institute, CB10 1SA, Hinxton, UK and ^3^Malawi-Liverpool-Wellcome Trust Clinical Research Programme, Box 30096 BT3, Blantyre, Malawia

## Abstract

**Summary:** Individuals living in endemic areas generally harbour multiple parasite strains. Multiplicity of infection (MOI) can be an indicator of immune status and transmission intensity. It has a potentially confounding effect on a number of population genetic analyses, which often assume isolates are clonal. Polymerase chain reaction-based approaches to estimate MOI can lack sensitivity. For example, in the human malaria parasite *Plasmodium falciparum*, genotyping of the merozoite surface protein (*MSP1/2*) genes is a standard method for assessing MOI, despite the apparent problem of underestimation. The availability of deep coverage data from massively parallizable sequencing technologies means that MOI can be detected genome wide by considering the abundance of heterozygous genotypes. Here, we present a method to estimate MOI, which considers unique combinations of polymorphisms from sequence reads. The method is implemented within the *estMOI* software. When applied to clinical *P.falciparum* isolates from three continents, we find that multiple infections are common, especially in regions with high transmission.

**Availability and implementation:** *estMOI* is freely available from http://pathogenseq.lshtm.ac.uk.

**Contact:** samuel.assefa@lshtm.ac.uk

**Supplementary information:** Supplementary data are available at *Bioinformatics* online.

## 1 BACKGROUND

Multiplicity of infection (MOI) refers to the number of different parasite genotypes co-infecting a single host. It is an epidemiological measure that can improve the understanding of many areas of parasitology, including the dynamics of infections, pathogenesis, effect of transmission intensity, drug efficacy and parasite genetics (Ross *et al.*, 2012). In malaria endemic areas, MOI can be a useful indicator of the transmission level, where the latter is positively correlated with the average number of malaria parasite strains in an individual (Babiker *et al.*, 1999). The merozoite surface proteins (MSPs) are involved in erythrocyte invasion and affect parasite density and eventually severe pathology. Genotyping of the *MSP2* gene is a standard method for assessing MOI in *Plasmodium falciparum* studies, as it is highly polymorphic in length and sequence (Ntoumi *et al.*, 1995). High-resolution genotyping of the *MSP2* gene can distinguish between infections by detecting the presence of different alleles at a polymorphic marker. However, the number of infections may not be accurately counted, as parasites from multiple infections may carry the same allele. Several methods have been developed using observed allele frequencies (Ross *et al.*, 2011), but they do not account for detectability. Polymerase chain reaction-based approaches struggle to detect parasites at low levels in samples, leading to model estimates referring only to infections that would have been counted if they had been distinguished in the genotyping. Approaches that consider the whole genome rather than candidate regions, and exploit the potentially high depth of coverage from sequencing technologies allowing detection of parasite at low levels, are likely to be more informative (Bowman *et al.*, 2013).

Massively parallelizable sequencing technologies are providing whole-genome data on various parasites including *P.falciparum* (haploid genome, 14 chromosomes, size 23 Mb, 19% GC content) isolates to high coverage depth (Robinson *et al.*, 2011). In this setting, the presence of heterozygous genotypes not only provides evidence of MOI but also complicates population genetic and diversity analysis. For example, the calculation of heterozygosity and detection of signatures of recent positive selection assume clonal samples, and *de novo* assembly of genomes derived from multiclone infections can lead to potentially cryptic gene characterizations. A common approach to overcome the problem of multiplicity is to culture the parasite in the laboratory to (near-) clonality, but this is time-consuming and not feasible for large numbers of field isolates. In addition, parasites sampled after a long-term culture will not fully represent the genotypes that are circulating in the population at the time of sampling due to clone loss and chromosomal deletions. Here, we adopt a sequence-based genome-wide approach to estimating MOI, which considers all possible local estimates based on combinations of alleles from read pairs. Being genome-wide, it is possible to identify genic regions of high multiplicity, thereby informing the development of new assays for inference. Our approach has been implemented in the *estMOI* software package.

## 2 ALGORITHM AND APPLICATION

The *estMOI* is a suite of Perl scripts that estimates the MOI locally in the genome and then overall to obtain a global estimate. The inputs are alignment (BAM files), variant regions in the Variant Call Format/VCF and an optional file of regions to exclude from analysis. Minimal multiplicity is inferred by considering the maximum number of distinct haplotypes formed by combinations of a user-specified number of single nucleotide polymorphisms (SNPs) on single or paired reads. The default setting is to consider SNPs on read pairs, as haplotypes formed using too many SNPs on only single reads, may lead to MOI artefacts due to sequencing errors. The local minimum haplotype frequency (default value 3) and total count (coverage, default 10) can be set to reduce the number of spurious estimates due to sequencing or mapping errors. In addition, a distributional cut-off can be set to exclude extreme values when estimating overall MOI (default 90th percentile).

To evaluate *estMOI*, it was implemented on several collections of *P.falciparum* with Illumina deep sequencing data. All the corresponding raw sequence data [SRA Study ERP000190, Manske *et al.* (2012); Robinson *et al.* (2011)] consists of paired end reads of minimum length 54 bp and was mapped to the 3D7 *P.falciparum* reference genome (v3.0) using *smalt* (www.sanger.ac.uk/resources/software/smalt/). Accuracy of MOI estimates using whole-genome sequence data may be affected by low read coverage, and all isolates had at least 30-fold coverage. The alignments were processed as described previously (Robinson *et al.*, 2011) to construct VCF (v4.1) files consisting of SNPs and indels (with quality scores of 30 or more). An exclusion file consisting of sub-telomeric and highly variable gene families was also constructed. The software was executed using default values, and the average run time per sample on standard desktop was ∼10 min, a process that is highly parallelizable.

The algorithm was tested on a resequencing data of the 3D7 reference genome, where as expected few SNPs were found, and an MOI of 1 was confirmed. To assess its performance on other clonal samples, we used sequence data from four isolates that have been under long-term culture [DD2, GB4, HB3 and 7G8, (Sepulveda *et al.*, 2013)]. The four clonal strains all had MOI of one. To evaluate *estMOI* in the case of mixed infections, we combined reads from two clonal isolates and confirmed a MOI of 2. As *estMOI* accuracy in a clinical sample setting may be affected by SNP density, we considered samples with at least 25K differences from the reference genome. We applied *estMOI* to sequence data for 54 clinical isolates from west Africa, where multiplicity information using *MSP1* and *MSP2* genotyping was available (Amambua-Ngwa *et al.*, 2012). The estimates for the presence of multiplicity from our method were in 65% concordant with MSP results. The 35% discordance arises when our method estimated the presence of multiple genotypes, whereas the MSP typing reported single infections. This difference may be explained by the high error rate of MSP-based genotyping and its potentially low detectability (Ross *et al.*, 2011).

To infer any relationship between the estimated MOI and transmission, we considered sequence data from Burkina Faso (n = 25, medium transmission), Cambodia (n = 25, low and cultured), Malawi (n = 25, medium to high), Mali (n = 25, medium), Thailand (n = 25, low and cultured) and Papua New Guinea (n = 11) (Preston *et al.*, 2012; M.Preston, submitted for publication). The MOI estimate for the clinical isolates varied according to transmission intensity and geographical origin of samples. Isolates from southeast Asia, where transmission is lower had the least proportion of multiple infections (Cambodia 4%; Thailand 7%, Papua New Guinea 16%). Conversely, isolates from Africa had a higher proportion of multiple infections (Mali 44%; Malawi 48%, Burkina Faso 54%). A previous study in Malawi estimated MOI from a single locus circumsporozoite protein gene using deep sequencing of T-cell epitope haplotypes (mean MOI 2.31) and genotyping of the NANP repeat region (mean MOI 1.29). Our Malawi result (mean MOI 3.47) is higher than the former, supporting the potential utility of our approach and whole-genome estimates.

## 3 DISCUSSION

The number of whole-genome sequenced parasite samples taken directly from malaria patients is growing rapidly, primarily due to improvements in sequencing technology, throughput and multiplexing. Establishing the MOI of *P.falciparum* samples using sequence data will assist with understanding the dynamics of infections, pathogenesis, effect of transmission intensity, drug efficacy and parasite genetics. The *estMOI* software can rapidly determine an estimate of MOI, and we have demonstrated that these results correlate highly with both MSP2 genotyping and transmission intensity. A sufficient density of SNPs is required to provide local estimates, but too great concentration of SNPs may be evidence of sequencing or mapping errors. Data filtering based on mapping qualities and coverage can assist with minimizing over-inflated MOI estimates. The *estMOI* may be used to identify potentially informative regions with high MOI across multiple samples as suitable candidates for future genotyping. We identified 26 potentially MOI informative genes (see Supplementary Materials). Further use may come from applying *estMOI* to other organisms, especially those that are highly polymorphic. The application to 26 publicly available *Plasmodium vivax* isolates (Auburn *et al.*, 2013) revealed multiple infections in 27% of the samples. In the future, it is expected that developments in single molecule sequencing are likely to increase read length and improve MOI estimates. However, in mixtures of highly related genomes, it may not be possible to accurately estimate the MOI, even when using whole-chromosome sequences (because they segregate independently) (Nkhoma *et al.*, 2012). Nonetheless, technological developments will increase the read length and accuracy of polymorphic calls, making our approach more robust and sensitive.

## Supplementary Material

Supplementary Data
