# Supplementary material for: estMOI: estimating multiplicity of infection using parasite deep sequencing data
Source: Bioinformatics. 2014 Jan 17;30(9):1292–4. doi: 10.1093/bioinformatics/btu005 (PMC3998131; doi:10.1093/bioinformatics/btu005)
Supplement: Supplementary Data [file supp_30_9_1292__index.html]

estMOI: estimating multiplicity of infection using parasite deep sequencing data — estMOI: estimating multiplicity of infection using parasite deep sequencing data — Supplementary Data 

# estMOI: estimating multiplicity of infection using parasite deep sequencing data

## Supplementary Data

files

**Files in this Data Supplement:**

- Supplementary Data - pdf file
