# Supplementary material for: estMOI: estimating multiplicity of infection using parasite deep sequencing data
Source: Bioinformatics. 2014 Jan 17;30(9):1292–4. doi: 10.1093/bioinformatics/btu005 (PMC3998131; doi:10.1093/bioinformatics/btu005)
Supplement: Supplementary Data [file supp_btu005_SupplementaryMaterials.pdf]

Supplementary Table 1: MOI estimates of two clonal samples (Sample 1 and Sample 2) and a mixed sample. The individual samples both had a MOI of one, where as the mixed sample had a MOI of 2. A minimum haplotype count of 3 and a percentile cutoff of 90 were used.

	#MOI	Counts	%	
**MIXED**	1	14356	0.71	
	**2**	**5076**	**0.96**	**MOI-estimate**
	3	618	0.99	
	4	79	1.00	
	5	28	1.00	
	>=6	8	1.00	
**SAMPLE1**	**1**	**9499**	**0.91**	**MOI-estimate**
	2	915	1.00	
	3	49	1.00	
	4	2	1.00	
	5	1	1.00	
	>=6	0	1.00	
**SAMPLE2**	**1**	**11812**	**0.91**	**MOI-estimate**
	2	971	0.99	
	3	120	1.00	
	4	30	1.00	
	5	6	1.00	
	>=6	0	1.00	

Supplementary Table 2: A list of 26 MOI informative genes (genes with high MOI across multiple sample). These genes could be used for future genotyping to estimate multiplicity in clinical *Plasmodium falciparum* samples.

**[Gene ID]**	**[Genomic Location]**
PF3D7_0211700	Pf3D7_02_v3: 469,790 - 473,491 (+)
PF3D7_0317300	Pf3D7_03_v3: 699,544 - 709,728 (-)
PF3D7_0403200	Pf3D7_04_v3: 178,518 - 180,200 (+)
PF3D7_0405300	Pf3D7_04_v3: 282,403 - 288,297 (-)
PF3D7_0410000	Pf3D7_04_v3: 464,732 - 467,339 (-)
PF3D7_0512800	Pf3D7_05_v3: 548,406 - 553,340 (-)
PF3D7_0605800	Pf3D7_06_v3: 237,811 - 244,521 (-)
PF3D7_0629700	Pf3D7_06_v3: 1,221,941 - 1,242,922 (+)
PF3D7_0710200	Pf3D7_07_v3: 463,105 - 471,837 (+)
PF3D7_0715200	Pf3D7_07_v3: 676,061 - 682,681 (-)
PF3D7_0723800	Pf3D7_07_v3: 993,232 - 1,000,233 (+)
PF3D7_0807400	Pf3D7_08_v3: 384,298 - 385,797 (+)
PF3D7_0827300	Pf3D7_08_v3: 1,181,295 - 1,183,796 (+)
PF3D7_1010400	Pf3D7_10_v3: 415,404 - 417,471 (-)
PF3D7_1131800	Pf3D7_11_v3: 1,227,220 - 1,231,950 (-)
PF3D7_1229100	Pf3D7_12_v3: 1,192,888 - 1,199,214 (-)
PF3D7_1240700	Pf3D7_12_v3: 1,728,774 - 1,730,007 (+)
PF3D7_1243700	Pf3D7_12_v3: 1,829,660 - 1,831,057 (+)
PF3D7_1322100	Pf3D7_13_v3: 920,971 - 929,449 (+)
PF3D7_1324300	Pf3D7_13_v3: 1,002,748 - 1,019,303 (-)
PF3D7_1329800	Pf3D7_13_v3: 1,265,137 - 1,268,763 (+)
PF3D7_1348400	Pf3D7_13_v3: 1,935,596 - 1,947,380 (-)
PF3D7_1422400	Pf3D7_14_v3: 900,386 - 905,278 (+)
PF3D7_1448000	Pf3D7_14_v3: 1,960,033 - 1,963,962 (-)
PF3D7_1472200	Pf3D7_14_v3: 2,945,662 - 2,952,551 (+)
PF3D7_1472600	Pf3D7_14_v3: 2,962,986 - 2,965,086 (-)
